# Dr. Adams on the Appearance of the Stomach after Death

**Published:** 1814-03

**Authors:** J. Adams

**Affiliations:** Hatton Garden


THE
### Medical and Physical Journal.

### DR. ADAMS *on the Appearance of the Stomach after Death.*

IN your last Number, two of your correspondents have done me the honor of noticing one of my papers: the principal matters between us being before the public, I shall confine myself to one or two points; but first permit me to repeat a sentence contained in my former paper. "All this will be considered as matter of conjecture, which I am sure those gentlemen will receive as offered to their further consideration." I am truly glad they have considered it, and though though not entirely according to my wishes, yet enough to keep alive the attention of us all to the importance of Mr. Hunter's doctrines.

But as every controversy must become tedious to your readers, I shall be careful not to occupy too much of your valuable pages. Let me first then in general remark that the whole of my paper, and all the opinions formerly offered by me, go on the suspicion that the common symptoms of hydrophobia arise from inflammation of the stomach—that I have admitted all the appearances urged by Mr. Abel in his last paper as imputable to inflammation, and have only cautioned those who examine stomachs after death not to trust to its vascular appearance as a proof of inflammation, when they find an *erosion*, or I might, perhaps, have said, an absolute disappearance of that part of the mucous coat which should have covered the red vessels.

---

The delay of a month has given me an opportunity of perusing Dr. Yelloly's paper in the volume just published by the Medico-Chirurgical Society. It abounds with so many useful facts on the appearances of the stomach, that it cannot fail to lead to the establishment of some certain rules by which we may in future be directed in examining that organ after death. I shall, therefore, only add a few hints, submitted to the consideration of such as have leisure to pursue the subject experimentally.

By Dr. Y.'s experiment on the dog destroyed a few hours after taking several grains of corrosive sublimate, we find that the true appearance of an inflamed stomach is such as has been admitted—great redness, with extravasation into the substance of the villous coat.

Inequalities in the thickness of the stomach ought, as Dr. Y. observes, to be distinguished from erosions. When erosions have occurred after the swallowing of arsenic, which is a very common incident, may not the animal have lived long enough for mortification to have taken place; and may not the mortified or dead part have been digested; and may not this account for Mr. Brodie's having never discovered gangrene in the stomach, though we know that arsenic so frequently produces that effect on other parts not protected by the external cuticle?

Dr. Y. adds to his observations some very candid remarks on "Mr. Hunter's valuable paper on digestion of the stomach after death." Both admit the thinness of the coat at its larger curvature; but Dr. Y. conceives it could not, in any of the cases he examined, arise from digestion, because the ends of the vessels were not eroded, as must in that case happen; and as Mr. Hunter describes by saying that "blood pressed pressed from the larger branches into smaller ones, passed out at the digested ends of the vessels, and appeared like drops on the inner surface. Such a degree of digestion, however, (continues Dr. Y.) must, I conceive, be very rare." Yet Mr. Hunter asserts, "there are few dead bodies in which the stomach is not digested at its greater end." Notwithstanding this apparent difference, I am persuaded that both are right. Mr. Hunter spoke of those bodies which are brought for dissection, of which the stomach is not examined till it is *dead*. All Dr. Yelloly's subjects were examined only a day or two after apparent death, that is, after respiration, the circulation of the blood, and all the other actions by which life is supported, had ceased so as never to be restored, yet before the constituent parts had lost their life, or even their powers of contraction. One proof of this is mentioned incidentally by Dr. Y. "It would appear," says he, "that there is a power capable of being exerted in the artery itself, which carries on the blood to the capillaries, or to the veins, after the further supply of fresh blood from the heart is stopped;" in other words, that those parts still retain their life, the contraction of the arteries still continuing after the circulation has ceased. How long the parts retain their life must depend on a variety of circumstances,—the season of the year, the mode of dying, the age, and even the peculiar properties of the animal; but as long as they retain life, they remain insoluble by the gastric juice.

It may be right here to mark the difference between erosion and perforation. The former to a certain degree is found in many stomachs, and in most if the body has been kept long enough before it is opened; the latter never occurs unless the subject was in previous health, and dies a violent or sudden death; and if it occurs at all, it is within a day or two after death. These experiments are easily tried on rabbits, in the manner I have described in another part of your Journal. J. ADAMS.

*Hatton Garden, Jan.* 20, 1814.

